# Diisopropyl­ammonium 4-meth­oxy­benzoate

**DOI:** 10.1107/S1600536811023762

**Published:** 2011-06-30

**Authors:** Bin Wei

**Affiliations:** a Ordered Matter Science Research Center, Southeast University, Nanjing 211189, People’s Republic of China

## Abstract

In the crystal structure of the title compound, C_6_H_16_N^+^·C_8_H_7_O_3_^−^, inter­molecular N—H⋯O hydrogen bonds link the cations and anions into arrangements consisting of two cations and two anions each.

## Related literature

For background to organic phase-transition materials, see: Fu *et al.* (2009 ▶); Rheinstädter *et al.* (2002 ▶); Wu *et al.* (2011 ▶).
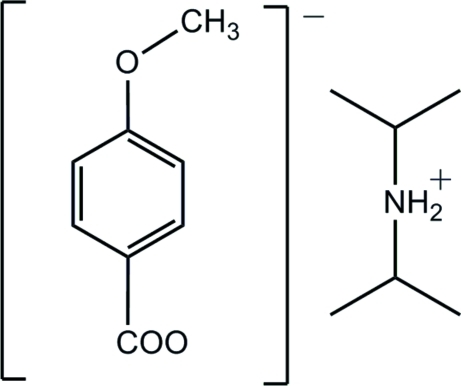


## Experimental

### 

#### Crystal data

C_6_H_16_N^+^·C_8_H_7_O_3_^−^
*M* *_r_* = 253.33
Triclinic, 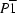

*a* = 7.3265 (15) Å
*b* = 8.8808 (18) Å
*c* = 12.107 (2) Å
α = 87.83 (3)°
β = 77.83 (3)°
γ = 83.44 (3)°
*V* = 764.9 (3) Å^3^
*Z* = 2
Mo *K*α radiation
μ = 0.08 mm^−1^
*T* = 293 K
0.20 × 0.20 × 0.20 mm

#### Data collection

Rigaku Mercury CCD diffractometer
Absorption correction: multi-scan (*CrystalClear*; Rigaku, 2005 ▶) *T*_min_ = 0.842, *T*_max_ = 1.000
7992 measured reflections
3501 independent reflections
1945 reflections with *I* > 2σ(*I*)
*R*_int_ = 0.027

#### Refinement

*R*[*F* ^2^ > 2σ(*F* ^2^)] = 0.058
*wR*(*F* ^2^) = 0.163
*S* = 1.04
3501 reflections
168 parameters
H-atom parameters constrained
Δρ_max_ = 0.14 e Å^−3^
Δρ_min_ = −0.20 e Å^−3^

### 

Data collection: *CrystalClear* (Rigaku, 2005 ▶); cell refinement: *CrystalClear*; data reduction: *CrystalClear*; program(s) used to solve structure: *SHELXS97* (Sheldrick, 2008 ▶); program(s) used to refine structure: *SHELXL97* (Sheldrick, 2008 ▶); molecular graphics: *SHELXTL* (Sheldrick, 2008 ▶); software used to prepare material for publication: *SHELXTL*.

## Supplementary Material

Crystal structure: contains datablock(s) I, global. DOI: 10.1107/S1600536811023762/im2287sup1.cif

Structure factors: contains datablock(s) I. DOI: 10.1107/S1600536811023762/im2287Isup2.hkl

Supplementary material file. DOI: 10.1107/S1600536811023762/im2287Isup3.cml

Additional supplementary materials:  crystallographic information; 3D view; checkCIF report

## Figures and Tables

**Table 1:** Hydrogen-bond geometry (Å, °)

*D*—H⋯*A*	*D*—H	H⋯*A*	*D*⋯*A*	*D*—H⋯*A*
N1—H1*B*⋯O3^i^	0.90	1.84	2.720 (2)	166
N1—H1*A*⋯O2^ii^	0.90	1.83	2.721 (2)	169

Symmetry codes: (i) 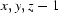; (ii) 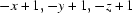.

**Fig. 1.**
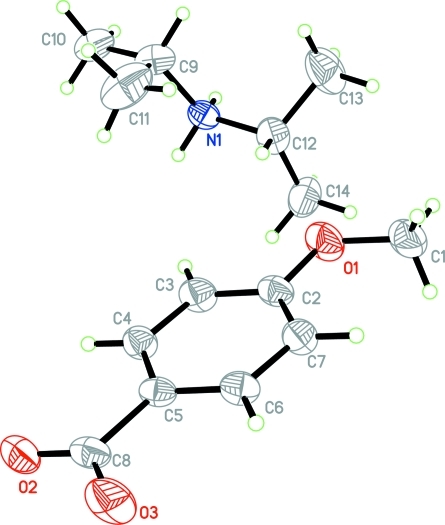
Molecular structure of the title compound, displacement ellipsoids are drawn at the 30% probability level.

**Fig. 2.**
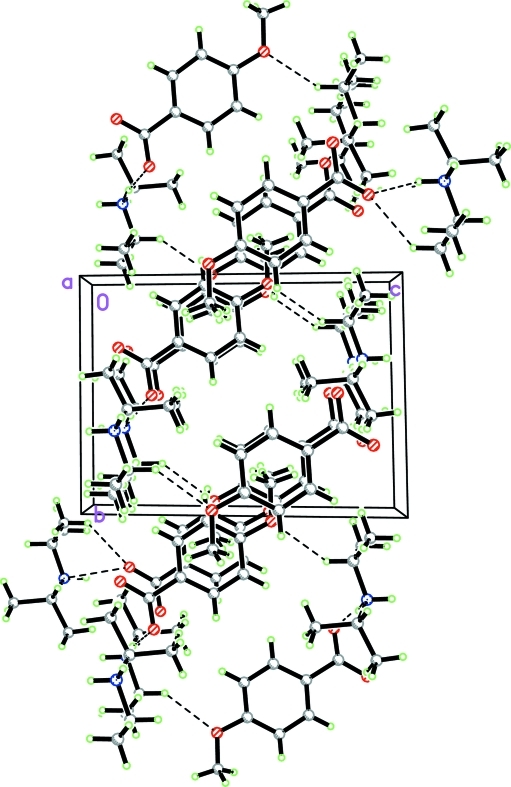
Packing of the title compound, stacking along the a axis. Dashed lines indicate hydrogen bonds.

## supplementary crystallographic information

### Comment

One of our main research topics is the search for new dielectric-ferroelectric materials. Recent studies have revealed that organic salts might also have this kind of character (Fu *et al.*, 2009; Rheinstädter *et al.*, 2002; Wu *et al.*, 2011). The dielectric constant of the title compound, diisopropylammonium 4-methoxy-benzoate, as a function of temperature indicates that the permittivity is basically temperature-independent. Below the melting point (438–440 K) of the title compound, we have found no dielectric disuniform from 80 K to 405 K. Herein we describe the crystal structure of this compound.

The asymmetric unit of the title compound consists of a diisopropylammonium cation and a 4-methoxybenzoate anion (Fig. 1). Intermolecular N—H···O hydrogen bonds (N1–H1A···O2 1.832 (2) Å, N1–H1B···O3 1.838 (2) Å) link the cations and anions into planar arrangements of two cations and anions each (Fig. 2 and Tab. 1).

### Experimental

The title compound was obtained by the addition of *para*-methoxybenzoic acid (1.52 g, 0.01 mol) to a solution of diisopropylamine (1.02 g, 0.01 mol) in methanol in a 1: 1 ratio. Good quality single crystals were obtained by slow evaporation of the solvent after two days (yield: 37%).

### Refinement

Positional parameters of all the H atoms bonded to C atom were calculated geometrically with C—H = 0.93 Å (CH_ar_), 0.98 Å (CH), 0.96 Å (CH_3_), with *U*_iso_(H) = 1.2 *U*_eq_(CH_ar_, CH) and *U*_iso_(H) = 1.5 *U*_eq_(CH_3_). Nitrogen bound H atoms were locatd in a difference Fourier map and refined freely

### Crystal data

**Table:**

C_6_H_16_N^+^·C_8_H_7_O_3_^−^	*V* = 764.9 (3) Å^3^
*M**_r_* = 253.33	*Z* = 2
Triclinic, *P*1	*F*(000) = 276
Hall symbol: -P 1	*D*_x_ = 1.100 Mg m^−^^3^
*a* = 7.3265 (15) Å	Mo *K*α radiation, λ = 0.71073 Å
*b* = 8.8808 (18) Å	θ = 6.2–55.3°
*c* = 12.107 (2) Å	µ = 0.08 mm^−^^1^
α = 87.83 (3)°	*T* = 293 K
β = 77.83 (3)°	Prism, colorless
γ = 83.44 (3)°	0.20 × 0.20 × 0.20 mm

### Data collection

**Table:**

Rigaku Mercury CCD diffractometer	3501 independent reflections
Radiation source: fine-focus sealed tube	1945 reflections with *I* > 2σ(*I*)
graphite	*R*_int_ = 0.027
Detector resolution: 28.5714 pixels mm^-1^	θ_max_ = 27.5°, θ_min_ = 3.0°
ω scans	*h* = −9→9
Absorption correction: multi-scan (*CrystalClear*; Rigaku, 2005)	*k* = −11→11
*T*_min_ = 0.842, *T*_max_ = 1.000	*l* = −15→15
7992 measured reflections	

### Refinement

**Table:**

Refinement on *F*^2^	Secondary atom site location: difference Fourier map
Least-squares matrix: full	Hydrogen site location: inferred from neighbouring sites
*R*[*F*^2^ > 2σ(*F*^2^)] = 0.058	H-atom parameters constrained
*wR*(*F*^2^) = 0.163	*w* = 1/[σ^2^(*F*_o_^2^) + (0.0667*P*)^2^ + 0.0936*P*] where *P* = (*F*_o_^2^ + 2*F*_c_^2^)/3
*S* = 1.04	(Δ/σ)_max_ < 0.001
3501 reflections	Δρ_max_ = 0.14 e Å^−^^3^
168 parameters	Δρ_min_ = −0.20 e Å^−^^3^
0 restraints	Extinction correction: *SHELXS97* (Sheldrick, 2008)
Primary atom site location: structure-invariant direct methods	Extinction coefficient: 0.0000

### Special details

**Table:**

Geometry. All e.s.d.'s (except the e.s.d. in the dihedral angle between two l.s. planes) are estimated using the full covariance matrix. The cell e.s.d.'s are taken into account individually in the estimation of e.s.d.'s in distances, angles and torsion angles; correlations between e.s.d.'s in cell parameters are only used when they are defined by crystal symmetry. An approximate (isotropic) treatment of cell e.s.d.'s is used for estimating e.s.d.'s involving l.s. planes.
Refinement. Refinement of *F*^2^ against ALL reflections. The weighted *R*-factor *wR* and goodness of fit *S* are based on *F*^2^, conventional *R*-factors *R* are based on *F*, with *F* set to zero for negative *F*^2^. The threshold expression of *F*^2^ > σ(*F*^2^) is used only for calculating *R*-factors(gt) *etc*. and is not relevant to the choice of reflections for refinement. *R*-factors based on *F*^2^ are statistically about twice as large as those based on *F*, and *R*- factors based on ALL data will be even larger.

### Fractional atomic coordinates and isotropic or equivalent isotropic displacement parameters (Å^2^)

**Table:**

	*x*	*y*	*z*	*U*_iso_*/*U*_eq_	
C5	0.3367 (2)	0.7243 (2)	0.69733 (13)	0.0464 (4)	
O2	0.4300 (2)	0.49545 (18)	0.78584 (12)	0.0787 (5)	
C2	0.2819 (3)	0.8953 (2)	0.50734 (15)	0.0535 (5)	
N1	0.2941 (2)	0.64829 (17)	0.11683 (11)	0.0526 (4)	
H1A	0.3909	0.5944	0.1403	0.063*	
H1B	0.3233	0.6510	0.0408	0.063*	
C3	0.3349 (3)	0.7417 (2)	0.49873 (15)	0.0610 (5)	
H3	0.3531	0.6951	0.4292	0.073*	
C6	0.2841 (2)	0.8778 (2)	0.70400 (14)	0.0533 (5)	
H6	0.2659	0.9246	0.7735	0.064*	
O1	0.2546 (2)	0.96828 (17)	0.40924 (11)	0.0763 (5)	
C8	0.3735 (3)	0.6323 (3)	0.79925 (16)	0.0570 (5)	
C7	0.2573 (3)	0.9648 (2)	0.60979 (16)	0.0572 (5)	
H7	0.2232	1.0688	0.6159	0.069*	
O3	0.3476 (2)	0.70132 (19)	0.89014 (11)	0.0877 (5)	
C4	0.3609 (3)	0.6570 (2)	0.59302 (15)	0.0541 (5)	
H4	0.3951	0.5531	0.5866	0.065*	
C12	0.2805 (3)	0.8068 (2)	0.15741 (17)	0.0678 (6)	
H12	0.2411	0.8061	0.2400	0.081*	
C1	0.2132 (3)	1.1280 (3)	0.4104 (2)	0.0807 (7)	
H1C	0.0956	1.1550	0.4615	0.121*	
H1D	0.2050	1.1645	0.3358	0.121*	
H1E	0.3110	1.1729	0.4347	0.121*	
C9	0.1253 (3)	0.5634 (3)	0.15227 (18)	0.0706 (6)	
H9	0.0195	0.6204	0.1260	0.085*	
C10	0.1664 (3)	0.4115 (3)	0.0961 (2)	0.0842 (7)	
H10A	0.2012	0.4256	0.0157	0.126*	
H10B	0.0565	0.3585	0.1142	0.126*	
H10C	0.2677	0.3533	0.1227	0.126*	
C13	0.1367 (4)	0.9106 (3)	0.1084 (2)	0.1088 (10)	
H13A	0.1710	0.9092	0.0274	0.163*	
H13B	0.1334	1.0121	0.1340	0.163*	
H13C	0.0150	0.8760	0.1329	0.163*	
C14	0.4734 (4)	0.8603 (3)	0.1257 (2)	0.0937 (8)	
H14A	0.5591	0.7954	0.1610	0.141*	
H14B	0.4676	0.9623	0.1507	0.141*	
H14C	0.5162	0.8570	0.0451	0.141*	
C11	0.0733 (4)	0.5482 (4)	0.2805 (2)	0.1119 (10)	
H11A	0.1810	0.5040	0.3084	0.168*	
H11B	−0.0262	0.4844	0.3013	0.168*	
H11C	0.0323	0.6466	0.3127	0.168*	

### Atomic displacement parameters (Å^2^)

**Table:**

	*U*^11^	*U*^22^	*U*^33^	*U*^12^	*U*^13^	*U*^23^
C5	0.0389 (9)	0.0635 (12)	0.0368 (9)	−0.0063 (8)	−0.0079 (7)	0.0001 (8)
O2	0.1004 (12)	0.0710 (10)	0.0741 (10)	−0.0030 (9)	−0.0451 (9)	0.0112 (8)
C2	0.0514 (11)	0.0669 (13)	0.0438 (10)	−0.0079 (9)	−0.0145 (8)	0.0084 (9)
N1	0.0586 (9)	0.0630 (10)	0.0373 (7)	−0.0025 (8)	−0.0151 (7)	0.0005 (6)
C3	0.0759 (14)	0.0706 (14)	0.0386 (10)	−0.0052 (11)	−0.0176 (9)	−0.0056 (9)
C6	0.0517 (11)	0.0665 (13)	0.0408 (10)	−0.0013 (9)	−0.0092 (8)	−0.0081 (8)
O1	0.0980 (11)	0.0798 (10)	0.0548 (8)	−0.0058 (8)	−0.0292 (8)	0.0167 (7)
C8	0.0519 (11)	0.0781 (15)	0.0445 (10)	−0.0100 (10)	−0.0173 (8)	0.0063 (9)
C7	0.0594 (12)	0.0556 (11)	0.0563 (11)	−0.0009 (9)	−0.0144 (9)	−0.0006 (9)
O3	0.1175 (13)	0.1062 (12)	0.0389 (8)	0.0002 (10)	−0.0225 (8)	0.0049 (8)
C4	0.0600 (11)	0.0559 (11)	0.0471 (10)	−0.0022 (9)	−0.0144 (8)	−0.0029 (8)
C12	0.0924 (16)	0.0619 (13)	0.0492 (11)	−0.0001 (12)	−0.0180 (11)	−0.0074 (9)
C1	0.0791 (16)	0.0799 (16)	0.0873 (16)	−0.0149 (13)	−0.0294 (12)	0.0331 (12)
C9	0.0568 (12)	0.0953 (17)	0.0628 (13)	−0.0142 (12)	−0.0156 (10)	−0.0068 (11)
C10	0.0764 (16)	0.0961 (18)	0.0887 (17)	−0.0278 (13)	−0.0257 (13)	−0.0091 (14)
C13	0.124 (2)	0.0868 (18)	0.108 (2)	0.0388 (17)	−0.0293 (18)	−0.0183 (15)
C14	0.116 (2)	0.0678 (15)	0.1050 (19)	−0.0272 (15)	−0.0328 (16)	0.0033 (13)
C11	0.109 (2)	0.157 (3)	0.0698 (16)	−0.069 (2)	0.0121 (14)	−0.0062 (16)

### Geometric parameters (Å, °)

**Table:**

C5—C6	1.374 (2)	C12—C13	1.520 (3)
C5—C4	1.387 (2)	C12—H12	0.9800
C5—C8	1.509 (2)	C1—H1C	0.9600
O2—C8	1.244 (2)	C1—H1D	0.9600
C2—O1	1.372 (2)	C1—H1E	0.9600
C2—C7	1.375 (3)	C9—C10	1.505 (3)
C2—C3	1.376 (3)	C9—C11	1.523 (3)
N1—C12	1.494 (2)	C9—H9	0.9800
N1—C9	1.499 (2)	C10—H10A	0.9600
N1—H1A	0.9000	C10—H10B	0.9600
N1—H1B	0.9000	C10—H10C	0.9600
C3—C4	1.379 (2)	C13—H13A	0.9600
C3—H3	0.9300	C13—H13B	0.9600
C6—C7	1.389 (3)	C13—H13C	0.9600
C6—H6	0.9300	C14—H14A	0.9600
O1—C1	1.417 (3)	C14—H14B	0.9600
C8—O3	1.249 (2)	C14—H14C	0.9600
C7—H7	0.9300	C11—H11A	0.9600
C4—H4	0.9300	C11—H11B	0.9600
C12—C14	1.511 (3)	C11—H11C	0.9600
			
C6—C5—C4	117.94 (16)	O1—C1—H1D	109.5
C6—C5—C8	121.22 (16)	H1C—C1—H1D	109.5
C4—C5—C8	120.81 (17)	O1—C1—H1E	109.5
O1—C2—C7	124.49 (18)	H1C—C1—H1E	109.5
O1—C2—C3	115.47 (17)	H1D—C1—H1E	109.5
C7—C2—C3	120.04 (17)	N1—C9—C10	108.59 (17)
C12—N1—C9	117.80 (16)	N1—C9—C11	110.65 (17)
C12—N1—H1A	107.9	C10—C9—C11	111.9 (2)
C9—N1—H1A	107.9	N1—C9—H9	108.5
C12—N1—H1B	107.9	C10—C9—H9	108.5
C9—N1—H1B	107.9	C11—C9—H9	108.5
H1A—N1—H1B	107.2	C9—C10—H10A	109.5
C2—C3—C4	120.11 (17)	C9—C10—H10B	109.5
C2—C3—H3	119.9	H10A—C10—H10B	109.5
C4—C3—H3	119.9	C9—C10—H10C	109.5
C5—C6—C7	121.78 (17)	H10A—C10—H10C	109.5
C5—C6—H6	119.1	H10B—C10—H10C	109.5
C7—C6—H6	119.1	C12—C13—H13A	109.5
C2—O1—C1	118.28 (16)	C12—C13—H13B	109.5
O2—C8—O3	125.35 (18)	H13A—C13—H13B	109.5
O2—C8—C5	117.80 (17)	C12—C13—H13C	109.5
O3—C8—C5	116.83 (19)	H13A—C13—H13C	109.5
C2—C7—C6	119.14 (18)	H13B—C13—H13C	109.5
C2—C7—H7	120.4	C12—C14—H14A	109.5
C6—C7—H7	120.4	C12—C14—H14B	109.5
C3—C4—C5	120.98 (18)	H14A—C14—H14B	109.5
C3—C4—H4	119.5	C12—C14—H14C	109.5
C5—C4—H4	119.5	H14A—C14—H14C	109.5
N1—C12—C14	108.13 (18)	H14B—C14—H14C	109.5
N1—C12—C13	111.18 (18)	C9—C11—H11A	109.5
C14—C12—C13	111.6 (2)	C9—C11—H11B	109.5
N1—C12—H12	108.6	H11A—C11—H11B	109.5
C14—C12—H12	108.6	C9—C11—H11C	109.5
C13—C12—H12	108.6	H11A—C11—H11C	109.5
O1—C1—H1C	109.5	H11B—C11—H11C	109.5

### Hydrogen-bond geometry (Å, °)

**Table:**

*D*—H···*A*	*D*—H	H···*A*	*D*···*A*	*D*—H···*A*
N1—H1B···O3^i^	0.90	1.84	2.720 (2)	166
N1—H1A···O2^ii^	0.90	1.83	2.721 (2)	169

Symmetry codes: (i) *x*, *y*, *z*−1; (ii) −*x*+1, −*y*+1, −*z*+1.
